# Evidence for functional expression of TRPM7 channels in human atrial myocytes

**DOI:** 10.1007/s00395-012-0282-4

**Published:** 2012-07-18

**Authors:** Yan-Hui Zhang, Hai-Ying Sun, Kui-Hao Chen, Xin-Ling Du, Bo Liu, Lik-Cheung Cheng, Xin Li, Man-Wen Jin, Gui-Rong Li

**Affiliations:** 1 Department of Medicine, Li Ka Shing Faculty of Medicine, The University of Hong Kong, Pokfulam, Hong Kong, China; 2 Department of Cardiac Surgery, Union Hospital, Tongji Medical College, Huazhong University of Science and Technology, Wuhan, China; 3 Department of Pharmacology, Tongji Medical College, Huazhong University of Science and Technology, Wuhan, China; 4 Department of Surgery, Li Ka Shing Faculty of Medicine, The University of Hong Kong, Pokfulam, Hong Kong, China; 5 Department of Physiology, Li Ka Shing Faculty of Medicine, The University of Hong Kong, L4-59, Laboratory Block, 21 Sassoon Road, Pokfulam, Hong Kong, China

**Keywords:** Human atrial myocytes, TRPM7 channels, TRPM4 channels, Atrial fibrillation

## Abstract

Transient receptor potential melastatin-7 (TRPM7) channels have been recently reported in human atrial fibroblasts and are believed to mediate fibrogenesis in human atrial fibrillation. The present study investigates whether TRPM7 channels are expressed in human atrial myocytes using whole-cell patch voltage-clamp, RT-PCR and Western blotting analysis. It was found that a gradually activated TRPM7-like current was recorded with a K^+^- and Mg^2+^-free pipette solution in human atrial myocytes. The current was enhanced by removing extracellular Ca^2+^ and Mg^2+^, and the current increase could be inhibited by Ni^2+^ or Ba^2+^. The TRPM7-like current was potentiated by acidic pH and inhibited by La^3+^ and 2-aminoethoxydiphenyl borate. In addition, Ca^2+^-activated TRPM4-like current was recorded in human atrial myocytes with the addition of the Ca^2+^ ionophore A23187 in bath solution. RT-PCR and Western immunoblot analysis revealed that in addition to TRPM4, TRPM7 channel current, mRNA and protein expression were evident in human atrial myocytes. Interestingly, TRPM7 channel protein, but not TRPM4 channel protein, was significantly increased in human atrial specimens from the patients with atrial fibrillation. Our results demonstrate for the first time that functional TRPM7 channels are present in human atrial myocytes, and the channel expression is upregulated in the atria with atrial fibrillation.

## Introduction

Recent progress in studies on transient receptor potential (TRP) channels has greatly improved our understanding of cellular physiology and pathophysiology in different mammalian systems. The superfamily of TRP channels comprises 28 cation-permeable channels expressed throughout the animal kingdom. They include seven subfamilies based on their structure homology: TRPC (canonical), TRPV (vanilloid), TRPM (melastatin), TRPP (polycystin), TRPML (mucolipin), TRPA (ankyrin), and TRPN (no- mechanopotential) [3, 8]. TRP channels are expressed in most non-excitable and excitable tissues and involved in many fundamental cellular functions such as Ca^2+^ signaling, contraction, proliferation, and cell death [9, 38]. Several types of TRP channel genes have recently been described in the heart of different species. They include TRPC5 [4], TRPC6 [27], TRPM4 [15], TRPP1 and TRPP2 [6], in human cardiac tissue and/or myocytes; TRPC3/6, TRPV2/4, TRPM3/7, and TRPP2 in mouse cardiac tissue and/or myocytes, and TRPC1/3, TRPC6, and TRPM4 in rat cardiac myocytes [24]. It was reported that mRNA and protein of TRPC1/3 channels were upregulated in human atrial tissue with atrial fibrillation and a goat model of atrial fibrillation [47]. A recent report showed that TRPC3 channels regulate rat cardiac fibroblast proliferation by controlling calcium entry [20].

Most studies on TRP channels in cardiac tissues from different species, however, only demonstrate the presence of genes and/or protein expression of these channels. The information regarding the current properties of the TRP channels in native human cardiac myocytes is scarce in published literature. Earlier studies reported single channel current of TRPM4 channels in mouse sino-atrial node cells [10], rat ventricular myocytes [16], and human atrial myocytes [15]. Gwanyanya and coworkers demonstrated that a Mg^2+^-inhibited, TRPM6/7-like current was present in rat and pig ventricular myocytes [18, 19]. A recent study has demonstrated that TRPM7 channels are expressed in human atrial fibroblasts, which mediate Ca^2+^ signals and confer fibrogenesis in humans with atrial fibrillation [13]. It is unknown whether TRPM7 channels are present in human atrial myocytes. In the present study, we are interested in determining whether the functional TRPM7 channels are expressed in human atrial myocytes with whole-cell patch voltage-clamp, RT-PCR, and Western immunoblotting analysis.

## Materials and Methods

### Human atrial myocyte preparation

Atrial myocytes were enzymatically isolated from specimens of human right atrial appendage obtained from patients undergoing coronary artery bypass grafting, valve repair or replacement, and the patient information is shown in Table 1. The experimental procedure for obtaining the human atrial tissue was approved by the Ethics Committee of the University of Hong Kong (UW-10-174) based on the patients’ consent. The human cardiac cell isolation procedure was adopted as described previously [31, 32]. The isolated human atrial myocytes were used for whole-cell patch voltage-clamp recording of membrane current and identifying gene expression of TRPM channels.

**Table 1:** Characteristics of the patients

	Sinus rhythm	Atrial fibrillation
*n* (m/f)	38 (25/13)	22 (15/7)
Age (years old)	55 ± 3.2	57 ± 4.6
CAD (*n*)	33	3
MVD (*n*)	4	18
AVD/MVD (*n*)	1	1
Hypertension (*n*)	15	4
LVEF (%)	61.7 ± 2.3	57.2 ± 2.1
LA (mm)	36.2 ± 1.2	49.7 ± 1.5
Medication (*n*)		
Digitalis	3	10
ACE inhibitors	14	5
β-blockers	20	2
Calcium channel blockers	11	4
Diuretics	10	14
Nitrates	9	2

*CAD* coronary artery disease, *MVD* mitral valve disease, *AVD* aorta valve disease, *LVEF* left ventricular ejection factor, *LA* left atrial size, *ACE* angiotensin-converting-enzyme

### Solution and chemicals

Tyrode solution contained (mM) NaCl 140.0, CsCl 5.0, MgCl_2_ 1.0, CaCl_2_ 1.8, 4-(2-hydroxyethyl)-1-piperazineethanesulfonic acid (HEPES) 10.0 and glucose 10.0 (pH adjusted to 7.3 with NaOH). Divalent-free solution was prepared by simply omitting the CaCl_2_ and MgCl_2_ from the standard solution. Nifedipine (3 μM) was included in the extracellular solutions to block L-type Ca^2+^ channels. Na^+^-free external solution was prepared by substituting NaCl with *N*-methyl-d-glucamine-chloride (NMDG-Cl) when external solutions used to study cation permeability contained 10 mM of Na^+^, K^+^, Mg^2+^ or Ca^2+^, added to the NMDG-Cl solution. The Mg^2+^-free pipette solution (for recording TRPM7 current) contained (mM) Cs-aspartate 110.0, CsCl 20.0, Na-phosphocreatine 5.0, HEPES 10.0, Cs-EGTA 5.0, GTP 0.1, and Na-ATP 5.0, pH adjusted to 7.2 with CsOH. The pipette solution (for recording TRPM4 current) contained (mM): Cs-aspartate 110.0, CsCl 20.0, MgCl_2_ 1.0, Na-phosphocreatine 5.0, HEPES 10.0, Cs-EGTA 0.05, GTP 0.1, and Mg-ATP 5.0, pH adjusted to 7.2 with CsOH.

All chemicals were purchased from Sigma-Aldrich Chemicals (St Louis, MO, USA). Stock solutions were made with dimethyl sulfoxide (DMSO) for 2-aminoethoxydiphenyl borate (2-APB, 100 mM), A23187 (10 mM). The stocks were divided into aliquots and stored at −20 °C. LaCl_3_ stock solution (100 mM) was made with distilled water.

### Electrophysiology

A small aliquot of the solution containing the isolated human atrial myocytes was placed in an open perfusion chamber (0.5 ml) mounted on the stage of an inverted microscope (Diaphot, Nikon, Japan). Myocytes were allowed to adhere to the bottom of the chamber for 10–20 min and superfused at ~2 ml/min with Tyrode solution. Only quiescent rod-shaped cells with clear cross-striations were used for electrophysiological recording.

Whole-cell currents were recorded as described previously [31, 32]. Borosilicate glass electrodes (1.2-mm OD) were pulled with a Brown-Flaming puller (model P-97, Sutter Instrument Co, Novato, CA, USA) and had tip resistances of 1.5–3 MΩ when filled with the pipette solution. A 3 M KCl-Agar bridge was used as the reference electrode. The tip potential was zeroed before the patch pipette contacted the cell. After a gigaohm seal was obtained by negative pressure, the cell membrane was ruptured by applying a gentle negative pressure to establish the whole-cell configuration. Series resistance (3–6 MΩ) was compensated by 50–80% to minimize voltage errors. Junction potentials (calculated 15.7 mV) between pipette and bath solutions were not corrected for the patch clamp recording. Membrane currents were measured using an EPC-10 amplifier and Pulse software (Heka Elektronik, Lambrecht, Germany). Command pulses were generated by a 12-bit digital-to-analog converter controlled by Pulse software. The obtained data were stored on an IBM PC computer for offline data analysis. All experiments were conducted at room temperature (22–23 °C).

### Reverse transcript polymerase chain reaction

The reverse transcript polymerase chain reaction (RT-PCR) was performed with a procedure described previously [30]. Briefly, the total RNA was isolated using the TRIzol method (Invitrogen) from human atrial myocytes then treated with DNase I (Promega, Madison, WI, USA). Reverse transcription (RT) was performed with RT system (Promega, Madison, WI, USA) protocol in 20 μl reaction mixtures. RNA (1 μg) was used in the reaction, and a combination of oligo (dT) and random hexamer promoters was used for the initiation of cDNA synthesis. After RT, the reaction mixture (cDNA) was used for polymerase chain reaction (PCR). The forward and reverse PCR oligonucleotide primers chosen to amplify the cDNA are listed in Table 1. PCR was performed by a Promega PCR system with *Taq* polymerase and accompanying buffers. The cDNA in 2 μl aliquots was amplified by a DNA thermal cycler (MyCycler; Bio-Rad, Hercules, CA, USA) in a 25 μl reaction mixture containing 1.0 thermophilic DNA polymerase reaction buffer, 1.25 mM MgCl_2_, 0.2 mM each deoxynucleotide triphosphate (dNTP), 0.6 μM of each forward and reverse primer, and 1.0 U of *Taq* polymerase under the following conditions: the mixture was annealed at 50–60 °C (1 min), extended at 72 °C (2 min), and denatured at 95 °C (45 s) for 30 cycles. This was followed by a final extension at 72 °C (10 min) to ensure complete product extension. The PCR products were electrophoresed through a 1.5% agarose gel, and the amplified cDNA bands were visualized by ethidium bromide staining. The bands were imaged by Chemi-Genius Bio-Imaging System (Syngene, Cambridge, UK).

### Western immunoblotting analysis

The related ion channel proteins were determined with Western immunoblotting analysis [22, 46]. The specimens of human right atrial appendage obtained from patients undergoing coronary artery bypass grafting or valve repair were frozen and stored at −80 °C. For Western immunoblotting analysis, the specimens were homogenized in ice-cold modified RIPA lysis buffer (50 mM Tris-Cl, pH 8, 150 mM NaCl, 1% Nonidet P-40 (NP-40), 0.5% sodium deoxycholate, 1% SDS) with a small tissue-mincer then sonicated to promote lysis. The samples were then centrifuged at 13,000 rpm at 4 °C for 30 min. The supernatants were collected, and protein concentration was determined with Bio-Rad protein assay. Lysates containing equal amounts of protein were mixed with SDS sample buffer and denatured at 95 °C for 5 min. Samples were electrophoresed on SDS-PAGE gels, and transferred onto nitrocellulose membranes. Subsequently, membranes were blocked with 5% non-fat dried milk (Bio-Rad) in TTBS (0.1% Tween-20) for 1 h at RT and incubated overnight at 4 °C with primary antibodies (mouse monoclonal anti-TRPM7, NeuroMab, USA; goat polyclonal anti-TRPM4, Santa Cruz Biotechnology, Inc.; goat polyclonal anti-GAPDH, Santa Cruz Biotechnology). The membranes were treated with goat anti-mouse or donkey anti-goat IgG-HRP antibody (1:5000, Santa Cruz Biotechnology) for 1 h at room temperature. Blots were developed with enhanced chemiluminescence (ECL, GE Healthcare, Hong Kong) and exposed on X-ray film (Fuji Photo Film GmbH). The film was scanned, imaged by a Bio-Imaging System (Syngene, Cambridge, UK), and analyzed via Gene Tools software (Syngene).

### Immunocytochemistry

Isolated human atrial myocytes were washed with IMDM medium (Sigma-Aldrich) and seeded on coverslips pre-coated with Laminin. After 4–8 h adhering, the cells were washed with PBS, and fixed with PBS containing 2% paraformaldehyde (PFA) for 20 min, and subsequently permeabilized with PBS containing 0.1% Triton X-100 for 3 min. The cells were incubated with blocking buffer (5% BSA in PBS) for 1 h after washing three times with PBS. The cells were incubated at 4 °C overnight with a primary antibody diluted in PBS containing 3% BSA, washed with PBS, and incubated with the fluorescence-labeled secondary antibody (Invitrogen) in PBS with 3% BSA in the dark at room temperature for 1 h. The coverslip was then washed four times with PBS and mounted with ProLong Gold Anti-fade Reagent (Invitrogen, Hong Kong, China) for durable visualization. The coverslip was then observed and captured with confocal microscopy (Olympus FV300, Tokyo, Japan).

### Statistical analysis

The data are expressed as mean ± SEM. Paired and/or unpaired Student’s *t* test were used as appropriate to evaluate the statistical significance of differences between two group means; ANOVA was used for multiple groups. Values of *P* < 0.05 were considered to be statistically significant.

## Results

### TRPM7-like current in human atrial myocytes

A previous report by Gwanyanya and colleagues demonstrated a TRPM6/7-like cation current in rat and pig ventricular myocytes [18] when a Mg^2+^-free pipette solution was used to record the membrane current. In this study, we used a modified K^+^- and Mg^2+^-free pipette solution to investigate whether TRPM7-like current is present in cardiac myocytes isolated from human atrial specimens.

**Fig. 1:**
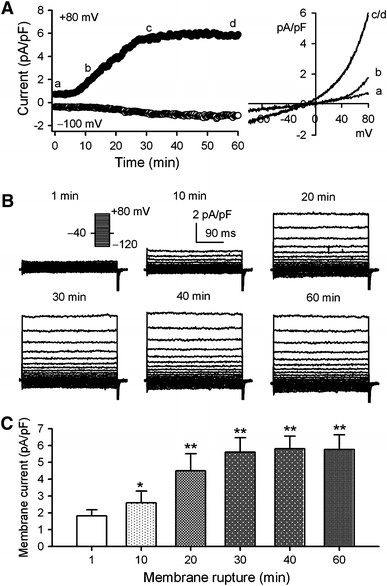
TRPM7-like current in human atrial myocytes. **a** Time course of membrane current recorded in a representative human myocyte with a K^+^ and Mg^2+^-free pipette solution using a 3-s ramp from −100 to +80 mV from a holding potential of −40 mV. Original ramp *I–V* currents at corresponding time points are shown in *right side* of the panel. **b** Voltage-dependent current traces recorded with 300-ms steps between −120 and +80 mV from a holding potential of −40 mV at 1, 10, 20, 30, 40, and 60 min after membrane rupture. **c** Mean values of the current at +80 mV at different time points of Mg_i_^2+^-free dialysis (*n* = 17, *P < 0.05, **P < 0.01 vs. 1 min)

Figure 1a illustrates the whole-cell current recorded with a 3-s voltage ramp from −100 to +80 mV from a holding potential of −40 mV in a representative human atrial myocyte. The membrane conductance gradually increased with the time of dialysis of the Mg^2+^-free pipette solution, and reached a steady-state level at 30–60 min after cell membrane rupture. Current–voltage (*I–V*) relationships were changed from linear to outward rectification with the Mg_i_^2+^-free dialysis. Voltage-dependent current elicited with 300 ms voltage steps between −120 and +80 mV from a holding potential of −40 mV (Fig. 1b) also exhibited an outward rectification. The step current showed rapid activation and outward rectification without inactivation as that described in HEK 293 cells stably expressing TRPM7 gene [25]. The increase of membrane conductance was observed in all the cells with Mg_i_^2+^-free dialysis, suggesting that TRPM7-like current is widely present in human atrial myocytes. The mean values of membrane current (+80 mV) at different time points of Mg_i_^2+^-free dialysis are illustrated in Fig. 1c. The membrane current density was significantly increased at 10-min dialysis and the steady-state level of the current was seen at 30–60 min dialysis (*n* = 17, *P* < 0.05 or *P* < 0.01 vs. 1 min dialysis).

**Fig. 2:**
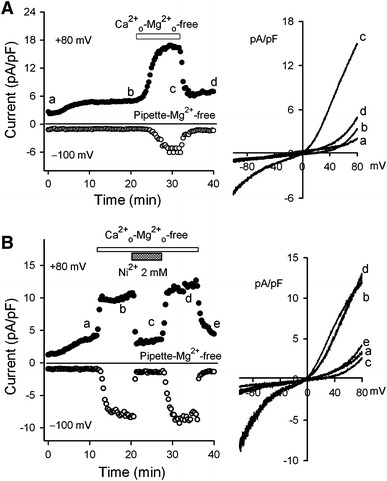
Enhancement of inward and outward currents by removing bath Ca^2+^ and Mg^2+^. **a** Time course of membrane current recorded in a typical experiment with the ramp protocol used in Fig. 1a with a K^+^- and Mg^2+^-free pipette solution. Removal of bath Ca^2+^ and Mg^2+^ (Ca_o_^2+^-Mg_o_^2+^-free) induced a remarkable increase of inward and outward currents. Original ramp *I–V* currents at corresponding time points are shown in right side of the panel. **b** Time course of membrane current recorded in another typical experiment with the ramp protocol used in Fig. 1a. The increased current induced by removing bath Ca^2+^ and Mg^2+^ (Ca_o_^2+^-Mg_o_^2+^-free) was inhibited by 2 mM Ni^2+^. Original ramp *I–V* currents at corresponding time points are shown in *right side* of the panel

Figure 2 displays that the removal of extracellular physiological divalent ions, e.g. bath Mg^2+^ and Ca^2+^, reversibly enhanced outward current and inward current (Fig. 2a), and application of 2 mM Ni^2+^ (Fig. 2b) or 2 mM Ba^2+^ (data not shown) inhibited the enhanced currents induced by removing extracellular Ca^2+^ and Mg^2+^. These properties are similar to those observed in pig and rat ventricular myocytes [18], suggesting that the current is likely mediated by TRPM7-like channels in human atrial myocytes.

**Fig. 3:**
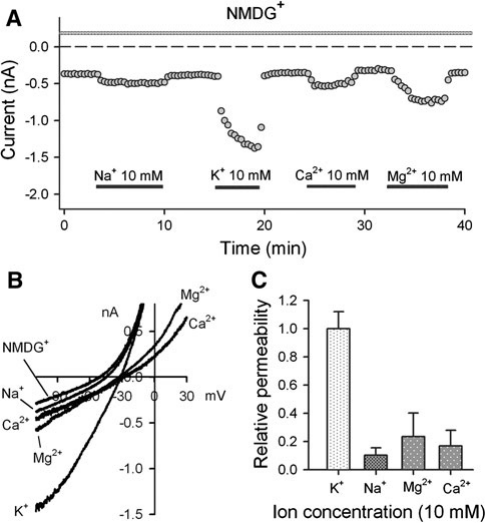
Permeability of TRPM7-like channels to physiological cations. **a** Time course of membrane inward current recorded in a representative cell with the ramp protocol used in Fig. 1a using a cation-free (NMDG) bath solution with application of equimolar (10 mM) Na^+^, K^+^, Mg^2+^, and Ca^2+^, respectively. **b** The original ramp inward currents in panel A with application of equimolar (10 mM) Na^+^, K^+^, Mg^2+^, and Ca^2+^, respectively. **c** Mean relative permeability of TRPM7-like channels to K^+^, Na^+^, Mg^2+^, and Ca^2+^ (*n* = 5–6) in human atrial myocytes

To examine ionic permeability of the TRPM7-like channels, physiological ions Na^+^, K^+^, Ca^2+^, and Mg^2+^ were applied at equimolar concentrations (10 mM) in Na^+^-free bath solution (Na^+^ was replaced by organic monovalent cation NMDG^+^) to examine their permeability through the TRPM7-like channels in human atrial myocytes. Figure 3 shows the ionic permeability of Na^+^, K^+^, Ca^2+^, and Mg^2+^. A large inward current was induced by 10 mM K^+^ and small inward current was induced by 10 mM Na^+^. Ca^2+^ or Mg^2+^ caused a small increase of inward current and significant inhibition of outward current (Fig. 3a, b). The mean permeability of physiological ions is illustrated in Fig. 3c. The K^+^ permeability was high, while the permeability to Na^+^, Mg^2+^, and Ca^2+^ was 0.10, 0.24, and 0.17 of K^+^, respectively. These results suggest that TRPM7-like channels are highly permeable to K^+^ in human atrial myocytes.

### Molecular identity of TRPM7-like current in human atrial myocytes

**Fig. 4:**
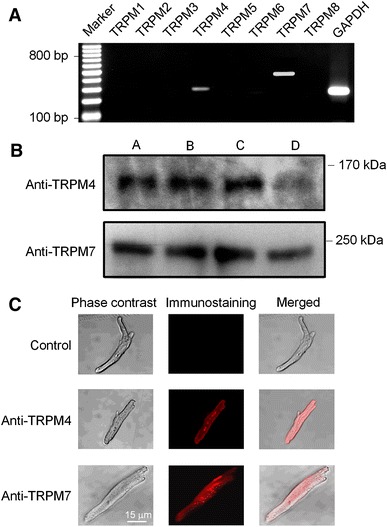
Gene and protein expression of TRPM channels in human atrial myocytes. **a** RT-PCR images for human TRPM channel genes. **b** Western immunoblots of TRPM4 and TRPM7 in human atrial tissues from four patients with sinus rhythm. **c** Immunostaining of TRPM4 and TRPM7 channels with anti-TRPM4 and anti-TRPM7 antibodies in human atrial myocytes

The molecular identity of TRPM7-like channels was determined with RT-PCR and Western immunoblot analysis. Figure 4a shows the results of RT-PCR using the primers designed for the human genes TRPM1, TRPM2, TRPM3, TRPM4, TRPM5, TRPM6, TRPM7, TRPM8, and GAPDH as shown in Table 2. The gene expression of TRPM4, TRPM7, and GAPDH was evident in human atrial myocytes. The expression of TRPM4 channels is consistent with the previous observation in human atrial myocytes [15]. RT-PCR showed that TRPM7 channel gene was also expressed in human atrial myocytes.

**Table 2:** Human gene-specific primers for RT-PCR

Gene (accession no.)	Primer sequences (5′–3′)	Fragment size (bp)
GAPDH (J_02642)	Forward AACAGCGACACCCACTCCTC Reverse GAGGGGAGATTCAGTGTGGT	258
hTRPM1 (NM_002420)	Forward TTTCGGACCCTTTACAAC Reverse TCTGCTCGTCATGCTTAT	421
hTRPM2 (NM_003307)	Forward ACGGACCAGATTTGGAAGTT Reverse ATGGCGTCAACCTTATTGC	299
hTRPM3 (NM_024971)	Forward TCATTATGCTGGTGGTTC Reverse AATATCATGGTCATGTGGC	436
hTRPM4 (NM_017636)	Forward GGCGGAGACCCTGGAAGACA Reverse CCAAGCCACAGCCAAACG	277
hTRPM5 (NM_014555)	Forward CTGGACGAGATTGATGAAGCC Reverse ACGAGCACCGAGCAGTAGTT	581
hTRPM6 (NM_017662)	Forward GACAACAGGAGCGTGGAT Reverse CAGGATGAAGTGCGAGTG	247
hTRPM7 (NM_017672)	Forward AAGCATTAGTTGCCTGTA Reverse GCATCTTGAGATTGTGGG	421
hTRPM8 (NM_024080)	Forward TGCCATCTCCTACGCTCTA Reverse TTCGCAACCAGTTTCCAG	372

Figure 4b shows the Western immunoblots of TRPM4 and TRPM7 proteins in human atrial specimens from four patients with sinus rhythm using anti-TRPM4 and anti-TRPM7 antibodies, respectively. The proteins of TRPM4 and TRPM7 channels were abundant in all human atrial tissues. Figure 4c illustrates the immunostaining of TRPM4 and TRPM7 expression in the surface of human atrial myocytes. These results confirm the expression of TRPM4 and TRPM7 channels in human atria, and the Mg^2+^-sensitive current observed above is mediated by TRPM7 channels.

### Effect of acidic pH on TRPM7 current in human atrial myocytes

**Fig. 5:**
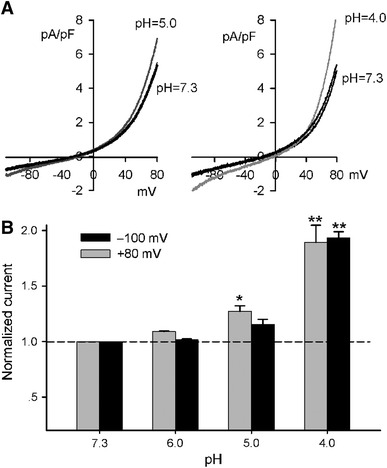
Effect of acidic bath pH on TRPM7 current in human atrial myocytes. **a** TRPM7 current recorded with the ramp protocol used in Fig. 1a at bath pH 7.3 and 5.0 (left panel), and pH 7.3 and 4.0 (*right panel*). **b** Normalized currents at −100 and +80 mV at different pH (*n* = 5, *P < 0.05, **P < 0.01 vs. pH 7.3)

Bath solution with acidic pH potentiated inward current in HEK 293 cells expressing TRPM7 and/or TRPM6 channels [33]. To examine how TRPM7 current in human atrial myocytes is affected by acidic bath pH, the membrane current was recorded by a 3-s ramp from −100 to +80 mV from a holding potential of −40 mV by altering bath pH from 7.3 to 6.0, 5.0, or 4.0 (Fig. 5). It is interesting to note that bath pH at 5.0 or 4.0 reversibly increased both inward and outward currents of TRPM7 in human atrial myocytes (Fig. 5a). The mean values of current alteration are illustrated in Fig. 5b. The current was not altered at pH 6.0, and significant potentiation of the current was observed when bath pH was lowered to 5.0 or 4.0 from 7.3. These results indicate that the sensitivity of TRPM7 in human atrial myocytes is similar, but not identical, to that observed in HEK 293 cells expressing TRPM7 and/or TRPM6 channels [33].

### Blockade of TRPM7 current by La^3+^ and 2-APB

**Fig. 6:**
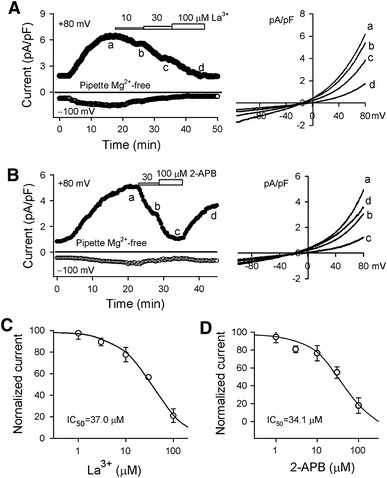
Blockade of TRPM7 current by La^3+^ or 2-APB. **a** Time course of membrane current recorded in a representative cell with K^+^- and Mg^2+^-free pipette solution using the ramp protocol used in Fig. 1a in the absence and presence of 10, 30, and 100 μM La^3+^. Original *I–V* traces at corresponding time points are shown in right side of the panel. **b** Time course of membrane current recorded in another typical experiment using the ramp protocol used in Fig. 1a in the absence and presence of 30 and 100 μM 2-APB. Original *I–V* traces at corresponding time points are shown in right side of the panel. **c** Concentration-dependent curve of La^3+^ for inhibiting the current at +80 mV was fitted to a Hill equation. **d** Concentration-dependent curve of 2-APB for inhibiting the current at +80 mV was fitted to the Hill equation

Figure 6 shows the effects of the non-specific TRPM7 channel blockers La^3+^ and 2-APB [5, 21, 33] on TRPM7 current in human atrial myocytes. The time course of TRPM7 current (Fig. 6a) recorded in a typical experiment with a 3-s ramp protocol (−100 to +80 mV from −40 mV) shows that the current was gradually increased by dialysis, and inhibited by La^3+^ at 10, 30 and 100 μM. IC_50_ (concentration for 50% inhibition) of La^3+^ for inhibiting TRPM7 current at +80 mV was 37.0 μM (Fig. 6c). Figure 6b displays the time course of TRPM7 current in another representative cell. 2-APB at 30 and 100 μM also reduced both inward and outward currents, and the reduction was partially reversed by washout. The IC_50_ of 2-APB for inhibiting TRMP7 current at +80 mV was 34.1 μM. These results indicate that both La^3+^ and 2-APB blocked TRPM7 current in human atrial myocytes like in other cell types [5, 21, 33].

### Whole-cell current of TRPM4 channels in human atrial myocytes

**Fig. 7:**
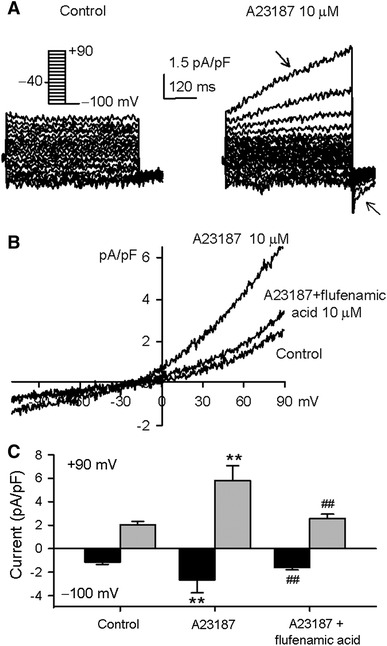
TRPM4 current in human atrial myocytes. **a** Voltage-dependent currents recorded in a typical experiment with 500-ms voltage steps between −100 to +90 mV from a holding potential of −40 mV before and after application of 10 μM A23187 (5 min). **b** Ramp *I–V* traces recorded in a typical experiments in the absence and presence of 10 μM A23187, and A23187 plus 10 μM flufenamic acid. **c** Mean values of the current measured at −100 and +90 mV before (control) and after 10 μM A23187 application, and A23187 plus 10 μM flufenamic acid (*n* = 7, **P < 0.01 vs. control; ^##^ *P* < 0.01 vs. A23187 alone)

A previous report characterized a single channel current of TRPM4, a Ca^2+^-activated non-selective cationic channel, in human atrial myocytes [15]. To demonstrate whole-cell current of TRPM4 channels in human atrial myocytes, we used a pipette solution with low EGTA (0.05 mM) to record membrane current with 500-ms voltage steps between −100 and +90 mV from a holding potential of −40 mV, and then back to −100 mV. Because TRMP4 channels are activated by intracellular free Ca^2+^ [15, 39], the Ca^2+^ ionophore A23187 [2] was used to activate the current. Figure 7a shows the voltage-dependent current traces in a representative myocyte before and after application of 10 μM A23187 to the bath solution. The time-dependent current with a large inward tail current at −100 mV, typical of TRPM4 current [39] was activated by application of A23187 for about 5 min. Similar results were obtained in four out of six myocytes treated with A23187.

In another group of experiments, we tested whether the TRPM4 inhibitor flufenamic acid [10] could inhibit the current. Figure 7b shows the *I–V* relationships of membrane current recorded in a typical experiment with a 3-s ramp protocol from −100 to +90 mV in the absence and presence of 10 μM A23187, and A23187 plus 10 μM flufenamic acid. A23187-activated current was significantly inhibited by flufenamic acid. In a total 12 myocytes, A23187 increased the membrane conductance in seven cells, and the increased current was antagonized by flufenamic acid (Fig. 7c, *n* = 7, *P* < 0.01 vs. A23187 alone). These results indicate that TRPM4 current is present in human atrial myocytes.

### TRPM4 and TRPM7 expression in human atria from patients with atrial fibrillation

**Fig. 8:**
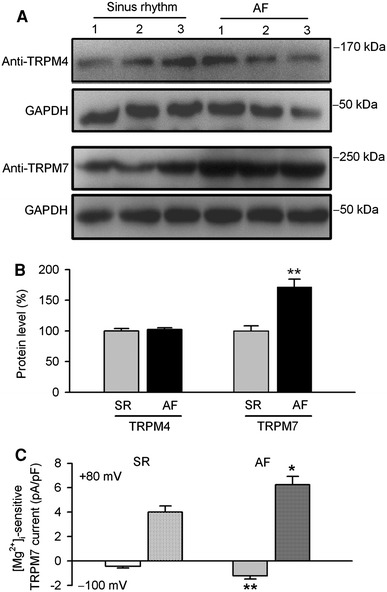
Alteration of TRPM channel protein expression in human atria from patients with atrial fibrillation (AF). **a** Western immunoblots of TRPM4 and TRPM7 in atria samples from patients with sinus rhythm (*n* = 3) or atrial fibrillation (*n* = 3). **b** Mean percentage values of relative expression of TRPM4 and TRPM7 channels in human atria from patients with sinus rhythm (SR) and atrial fibrillation (*n* = 12 patients for each group, **P < 0.01 vs. sinus rhythm). **c** Mean values of Mg_i_^2+^-free sensitive TRPM7 current obtained by digital subtraction of the current recorded at 30 min by the current recorded at 1 min of dialysis (*n* = 14 experiments from three AF patients, *P < 0.05, **<0.01 vs. SR cells, *n* = 18 experiments from five SR patients)

We finally determined whether TRPM4 and TRPM7 channel protein expression would be altered in atria from patients with atrial fibrillation. Figure 8a shows the Western immunoblot detection of TRPM4 and TRPM7 in atrial protein samples from three patients with sinus rhythm and three patients with atrial fibrillation. No significant change was observed in TRPM4 protein, while remarkable increase was observed in TRPM7 protein in the three atrial samples from patients with atrial fibrillation. Figure 8b displays the mean percentage values of TRPM4 and TRPM7 protein expression in the atria with sinus rhythm (*n* = 12) and the atria with atrial fibrillation (*n* = 12). TRPM7 channel protein expression was increased by 71.5 ± 12.8% in atria of patients with atrial fibrillation (*P* < 0.01 vs. sinus rhythm), while no change was seen for TRPM4 channel protein expression in atria of patients with atrial fibrillation (*P* = NS).

To examine whether TRPM7 current is upregulated in human atrial myocytes with atrial fibrillation, Mg_i_^2+^-sensentive current was analyzed in sinus rhythm myocytes and atrial fibrillation myocytes. Figure 8c illustrates the mean values of Mg_i_^2+^-sensentive TRPM7 current at −100 and +80 mV at 30 min Mg^2+^-free dialysis in human atrial myocytes without or with atrial fibrillation. The density of Mg_i_^2+^-sensentive TRPM7 current was greater in myocytes with atrial fibrillation than that in cells with sinus rhythm. This indicates an increase of TRPM7 current in human atrial fibrillation.

## Discussion

The present study demonstrates that both TRPM4 channels and TRPM7 channels are present in human atrial myocytes. TRPM7 current is gradually activated by dialysis with a Mg^2+^-free pipette solution, enhanced by removing bath divalent cations, and potentiated by acidic pH. TRPM7 channels are preferentially permeable to K^+^, and less permeable to equimolar (10 mM) Na^+^, Mg^2+^ and Ca^2+^. TRPM7 current is inhibited by La^3+^ and 2-APB. Whole-cell current of TRPM4 channels is activated by the Ca^2+^ ionophore A23187 in human atrial myocytes. Molecular identities of TRPM4 and TRPM7 channels are confirmed by RT-PCR, Western blot, and immunocytochemistry. Interestingly, TRPM7, but not TRPM4, is markedly upregulated in human atria from patients with atrial fibrillation.

More than ten TRP channels have been demonstrated to be expressed in the heart and vasculature of mammals including humans [23, 50]. They include TRPC1, 2, 3, 4, 6, and 7 channels in mouse sinoatrial node and/or myocytes [26], TRPC3/6, TRPV2, 4, TRPM3, 7, and TRPP2 channels in mouse cardiac tissue and/or myocytes and TRPC1, 3, and TRPC6 channels in rat cardiac myocytes [24], TRPC5 channels in human cardiac hypertrophic ventricle [4], TRPM4 channels in mouse sinus node cells [10], rat ventricular myocytes [17], and human atrial myocytes [15]. A recent study demonstrated that TRPC1, 4, 6, TRPV2, 4, and TRPM4, 7 channels are expressed in human atrial fibroblasts [13]. Although the molecular identity has not been confirmed, TRPM6/7-like channel current was described in rat and pig ventricular myocytes [18, 19]. The present study provides the novel information that in addition to TRPM4 channels, functional TRPM7 channels are expressed in human atrial myocytes.

TRPM7 (ChaK1, TRP-PLIK, LTRPC7) is a ubiquitous, calcium-permeant ion channel that is unique in being both an ion channel and a serine/threonine kinase regulated by intracellular ATP and phosphatidylinositol 4,5-bisphosphate (PIP_2_) [36, 43, 44]. The current is characterized by gradual activation during dialysis with Mg^2+^-free pipette solution, and is potentiated by removing bath divalent ions, increased by acidic pH, and inhibited by 2-APB and/or La^3+^ (or Gd^3+^) [13, 25, 33, 43].

In human atrial myocytes, as well as in pig and rat ventricular myocytes [18], the TRPM7 current was gradually activated by dialysis with Mg^2+^-free pipette solution. The inward and outward currents were potentiated by removing bath Ca^2+^ and Mg^2+^, and the potentiation effect was inhibited by Ni^2+^ or Ba^2+^. TRPM7 channels in human atrial myocytes are permeable to K^+^, Na^+^, Mg^2+^, and Ca^2+^, similar to the TRPM7 channels expressed in CHO cells [43]. Interestingly, TRPM7 current in human atrial myocytes was increased by acidic bath pH, similar to TRPM7 channels expressed in HEK 293 cells or TRPM7-like current in human atrial fibroblasts [13, 25, 33]. However, the response to bath pH is different from the TRPM6/7-like current observed in pig and rat ventricular myocytes, which was reduced by acidic bath pH [18]. It is unclear why the response of TRPM7 current in human atrial myocytes to acidic bath pH is opposite to the TRPM6/7-like current observed in pig and rat ventricular myocytes.

In addition, TRPM7 current in human atrial myocytes was inhibited by La^3+^ and 2-APB. The IC_50_ (34.1 μM) of 2-APB for inhibiting the current is close to that for inhibiting TRPM7 current in human atrial fibroblasts [13]. All properties described above support the notion that TRPM7 channels are expressed in human atrial myocytes. Moreover, the abundant expression of mRNA and protein further confirms the presence of TRPM7 channels in human atrial myocytes. However, the abundant protein of TRPM7 was measured in human atrial tissue, which could also be related to fibroblasts.

The upregulation of TRP channels is believed to mediate the progression of electrical remodeling and the arrhythmogenesis of the diseased heart [24]. TRPC3 expression is up-regulated in multiple rodent pathological cardiac hypertrophy models [4, 42]. TRPC-derived accumulation of intracellular Ca^2+^ is believed to contribute to selective activation of calcineurin in diseased heart [4]. Single channel activity and mRNA expression of Ca^2+^-activated TRPM4 channels were upregulated in ventricular myocytes of spontaneously hypertensive rats, and believed to be the cause of the delayed-after-depolarizations observed during intracellular Ca^2+^ overload of cardiomyocytes [17]. The present evidence for TRPM4 is consistent with previous observation for Ca^2+^-activated TRPM4 channels in human atrial myocytes [15]. However, it is unknown whether there is any potential contribution of TRPM4 channels to atrial fibrillation since no change in channel protein expression was observed in atrial tissues from patients with atrial fibrillation.

One of limitations of the present study was that the current was recorded with Cs^+^ to block potassium channels, and nifedipine to block calcium channels, but not 4,4′- Diisothiocyano-2,2′-stilbenedisulfonic acid (DIDS) to block chloride channels, and the current may be contaminated by chloride current, especially when the Ca^2+^ ionophore A23187 [2] was used to activate TRPM4 channels. Our earlier studies demonstrated that only volume-sensitive chloride current was recorded with hypotonic (0.6 T) insult, and no evidence for cAMP/protein kinase A-regulated chloride current or Ca^2+^-activated chloride current was observed in human atrial myocytes [28, 29]. Therefore, the potential contamination of the TRP current by chloride current would be very limited under physiological isotonic conditions in human atrial myocytes. On the other hand, there is no selective chloride channel blocker commercially available. The chloride channel blocker DIDS may also block TRPM4 channels [35].

Another limitation is that the atrial specimens were collected from a relative young patient population in the present study and only four patients with mitral valve disease in sinus rhythm group. The previous study established that valvular heart disease extensively remodels cardiac ion channel and transporter expression [14]. Nonetheless, the difference in patient age and heart disease would not affect the main outcome of the present study for demonstrating the evidence of TRPM7 channels in human atrial myocytes.

TRPM7 is a Ca^2+^ permeable channel, which is constitutively activated and brings Ca^2+^ into cells under physiological conditions [36, 43]. Although a previous study reported a TRPM6/7-like current in pig and rat ventricular myocytes and the presence of TRPM7 channels was demonstrated in human atrial myocytes in this study, further effort is required to determine the potential role of TRPM7 current in cardiac cellular biology and electrophysiology. Previous studies reported that oxidative stress, membrane stretch, and shear stress could activate TRPM7 [1, 40, 41], which may imply a potential role of TRPM7 in myocardial pathological process. A recent study has reported that TRPM7 channel gene expression is remarkably increased in fibroblasts isolated from human atria of patients with atrial fibrillation, it is, therefore, believed that TRPM7-mediated Ca^2+^ signals may mediate fibrogenesis in human atrial fibrillation [13].

In addition, abnormal Ca^2+^ signaling has been demonstrated to play an important role in atrial fibrillation pathophysiology [7, 12, 34, 45, 49]. A recent study in human atrial myocytes from atrial fibrillation patients clearly showed that Ca^2+^ handling changes are involved in atrial arrhythmogenesis, and delayed afterdeplorization-mediated triggered activity is more frequent in atrial myocytes from patients with atrial fibrillation [48]. It is believed that Ca^2+^-related functions in electrical and structural remodeling processes participate in leading to the atrial fibrillation substrate [37]. Therefore, enhanced TRPM7 channels may also participate in the higher propensity to delayed afterdepolarization-mediated ectopic activity in patients with chronic atrial fibrillation. The identification of new molecular targets is important for improving drug therapy and discovery of biomarkers for risk stratification, and is a major goal in the management of atrial arrhythmias [11].

In summary, the present study has demonstrated for the first time that in addition to Ca^2+^ TRPM4 current, TRPM7 current is present in human atrial myocytes. TRPM7, but not TRPM4, is upregulated in atria of individuals with atrial fibrillation. More effort is required to clarify whether and how TRPM7 channels in atrial myocytes mediate the initiation and/or maintenance of atrial fibrillation.
